# The Analysis of Fennel (*Foeniculum vulgare* Mill.) Chemical Composition by UPLC‐Q‐Exactive‐Plus‐Orbitrap‐MS and GC‐MS/MS

**DOI:** 10.1155/ianc/1248684

**Published:** 2026-10-06

**Authors:** Nier Wu, Junni Qi, XinXin Jin, Chula Sa, Yan Zhang

**Affiliations:** ^1^ College of Mongolian Medicine Inner Mongolian Medical University 010110 Hohhot China immu.edu.cn; ^2^ Department of Rehabilitation Affiliated Hospital of Inner Mongolia Medical University Inner Mongolia Medical University 010050 Hohhot China immu.edu.cn

**Keywords:** aqueous extract, chemical composition, *Foeniculum vulgare* Mill., GC-MS/MS, UPLC-Q-Exactive-plus-Orbitrap-MS, volatile oil

## Abstract

**Background:**

Fennel (*Foeniculum vulgare* Mill.) is widely used as a functional food and plays an important role in traditional medicine worldwide. However, a systematic analysis of its chemical components remains limited.

**Objective:**

This study aimed to comprehensively identify and analyze the chemical composition of both polar and nonpolar fractions of fennel, specifically its aqueous extracts and volatile oils.

**Methods:**

Polar metabolites, including phenolic acids, flavonoids, and coumarins, were profiled using ultra‐performance liquid chromatography coupled with quadrupole‐Orbitrap high‐resolution mass spectrometry (UPLC‐Q‐Exactive‐plus‐Orbitrap‐MS), while gas chromatography–tandem mass spectrometry (GC‐MS/MS) was employed for the analysis of volatile constituents.

**Results:**

A total of 87 compounds were identified in the fennel aqueous extract, including 10 flavonoids, 11 phenolic acids, 3 coumarins, 1 stilbene, 14 alkaloids, 13 organic acids, 7 fatty acids, 10 amino acids, 6 nucleosides, 2 glycosides, 3 terpenoids, and 6 other components (containing 1 sugar alcohol). In addition, 13 compounds were identified in the fennel volatile oil using n‐hexane and ethyl acetate as solvents, comprising 11 monoterpenes and 2 phenylpropanoids.

**Conclusion:**

These findings expand our understanding of the chemical composition of fennel and provide a foundation for elucidating its pharmacodynamic properties.

**Highlights**
1. This study reports, for the first time, the simultaneous analysis of the aqueous extract and volatile oil of *Foeniculum vulgare* Mill. using UPLC‐Q‐Exactive‐plus‐Orbitrap‐MS and GC‐MS/MS. In addition, the volatile oil constituents extracted using two different solvents (n‐hexane and ethyl acetate) were compared.
2. This is not only a comprehensive and systematic composition analysis of *Foeniculum vulgare* Mill. but also provides a basis for its development and utilization.

## 1. Introduction

Fennel (*Foeniculum vulgare* Mill.) is a perennial aromatic herb belonging to the Apiaceae family (Figure 1). Although the seeds (fruits) are the primary medicinal parts, the bulbs, leaves, and stems also possess therapeutic value. Native to the Mediterranean region, fennel is now widely cultivated globally, with India and China being major producers [1]. Renowned for its anise‐like aroma and sweet flavor, it is extensively used as a culinary spice and flavoring agent in the food, beverage, and confectionery industries, as well as in perfumes and cosmetics [1–4].

**FIGURE 1:**
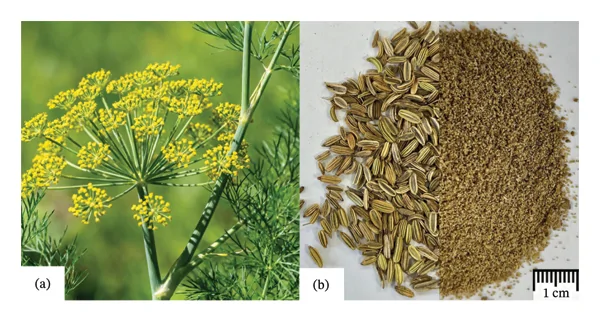
Plant and medicinal herbs of fennel: (a) plant diagram and (b) medicinal materials diagram (seed) and powder.

For centuries, fennel has been used both as a culinary spice and as a traditional herbal medicine in various ethnomedicinal systems [5]. It exhibits distinct therapeutic applications in traditional Chinese medicine (TCM), Ayurveda, traditional Mongolian medicine, and traditional Iranian medicine [6]. In TCM, it is characterized as warm and pungent‐sweet, with functions to regulate *qi*, harmonize the stomach, relieve pain, and disperse cold. It is commonly used to treat digestive and respiratory disorders as well as menstrual complaints [6, 7]. Ayurvedic medicine employs it as a carminative, stomachic, and galactagogue for gastrointestinal and respiratory ailments [6, 8]. In Mongolian medicine, it is used to treat “heyi” heat, blurred vision, toxic vomiting, epigastric distension, and food stagnation [9]. European traditional medicine utilizes it primarily for digestive complaints, as an expectorant, diuretic, and emmenagogue [6]. Modern pharmacological research has demonstrated that fennel exhibits a broad spectrum of biological activities, including antioxidant, antimicrobial, anti‐inflammatory, antitumor, cardioprotective, hepatoprotective, hypolipidemic, hypoglycemic, and neuroprotective properties [10, 11].

These medicinal benefits are attributed to its rich and diverse chemical composition. Fennel contains volatile oil (2%–4%), fixed oil, proteins, carbohydrates, minerals, vitamins, and various phytochemicals [2, 11]. The volatile oil, responsible for its characteristic aroma and many pharmacological activities, is the most extensively studied component, with *trans*‐anethole constituting 60%–90% [12]; other major constituents include fenchone, estragole, limonene, and α‐pinene [13]. Research has predominantly focused on the volatile oil fraction, whereas aqueous extract components such as phenolic acids, flavonoids, and coumarins have received limited attention. Previous studies using HPLC/UV‐ESI‐MS identified 28 compounds in fennel aqueous extract, with phenolic acids (49.96%), flavonoids (24.4%), and polyphenols (11.2%) as the main classes; key monomers included chlorogenic acid, dihydrocaffeic acid, and flavonoid glycosides such as quercetin‐3‐glucuronide and rutin [14–16]. Other investigations detected ribitol, phthalic acid, coumarins, terpenoids, alkaloids, amino acids, and nucleosides in aqueous extracts, which exhibited significant antioxidant and antibacterial activities [5, 17]. Notably, the extraction method substantially affects the composition, with water extraction and decoction favoring the dissolution of phenolic acids.

Despite these advances, no study has simultaneously employed ultra‐performance liquid chromatography coupled with quadrupole‐Orbitrap high‐resolution mass spectrometry (UPLC‐Q‐Exactive‐plus‐Orbitrap‐MS) and gas chromatography–tandem mass spectrometry (GC‐MS/MS) for the comprehensive analysis of both aqueous extract and volatile oil of F. vulgare. Therefore, we established an integrated analytical platform combining UPLC‐Q‐Exactive‐plus‐Orbitrap‐MS for polar nonvolatile constituents and GC‐MS/MS for volatile components. This approach aims to achieve complete chemical characterization, thereby supporting rational quality control and pharmacological research of this traditional medicinal and edible plant.

## 2. Materials and Methods

### 2.1. Instrument

Q Exactive Plus Orbitrap high‐resolution LC‐MS (Thermo Fisher), U3000 ultra‐high‐performance liquid chromatography system and autosampler (Thermo Fisher), ACQUITY UPLC HSS T3 column (2.1 × 100 mm, 1.8 μm; Waters Corporation), 7890B‐7000D GC‐MS/MS instrument (Agilent Technologies, Santa Clara, CA, USA), HP‐5MS capillary column (30 m × 0.25 mm × 0.25 μm), Vortex 2 Genie Vortex Mixer (Scientific Industries), 5810R Low temperature centrifuge (Eppendorf), WD‐9415C ultrasonic cleaner (Beijing Liuyi Instrument Factory), AL 204 Electronic Analytical Balance (Mettler‐Toledo, USA), and KQ‐250 Ultrasonic Cleaner (Gong Yuhua Instrument Co., Ltd).

## 3. Drugs and Reagents

### 3.1. Drugs

Fennel was purchased from the Anguo Runde Pharmaceutical Co., Ltd in 2024 (batch no. C24082604, production date: 20250410; place of origin: Gansu Province, China). The sample was authenticated as the mature fruit of *Foeniculum vulgare* Mill. by Professor Tumen Bayar (Teaching and Research Department of Mongolian Medicine, College of Mongolian Medicine, Inner Mongolia Medical University). Quality assessment was performed in accordance with the Chinese Pharmacopoeia (2025 edition), and the sample met all required specifications.

### 3.2. Reagents

C_8_‐C_40_ n‐alkane mixed reference standard was purchased from Tanzhi Quality Inspection (batch no. 24120110). Methanol and formic acid were purchased from Thermo Fisher (Pittsburgh, PA, USA). Deionized water was produced using the Milli‐Q Advantage A10 ultrapure water machine. n‐Hexane was purchased from Tianjin Beilian Fine Chemicals Development Co., Ltd. (batch no. 20220620). Ethyl acetate was purchased from Sinopharm Chemical Reagent Co., Ltd. (batch no. 20160321).

## 4. Sample Solution Preparation

### 4.1. Preparation of Aqueous Extract

Fennel powder (15 g) was placed in a beaker and extracted with 200 mL of distilled water by decoction. The mixture was boiled and maintained for 30 min with constant stirring. The solution was filtered through gauze to collect the decoction. The marc was re‐extracted twice with an equivalent volume (200 mL) of distilled water under identical conditions. The combined filtrates were subjected to suction filtration and subsequently dried completely at 80°C, yielding 23.5% extract (w/w, calculated based on the dry weight of raw material).

For qualitative analysis, fennel aqueous extract was accurately weighed and extracted with 1 mL of 80% (v/v) methanol. The mixture was sonicated for 10 min and then centrifuged at 14,000 rpm for 10 min. The supernatant (0.8 mL) was transferred to a clean centrifuge tube and centrifuged again. The resulting supernatant was transferred to an autosampler vial for UPLC‐Q‐Exactive‐plus‐Orbitrap‐MS analysis.

### 4.2. Extraction of Volatile Oil

Fennel powder (20 g) was placed in a round‐bottom flask, mixed with 10 volumes of water, soaked, and then gently heated to boiling. The mixture was maintained at a gentle boil for a specified duration. After heating was stopped, the mixture was cooled, and the volatile oil was collected to determine its yield. The yield was 4.5%.

The extracted volatile oil was collected, dried with anhydrous sodium sulfate, and then stored in the refrigerator at 4°C. An aliquot (10 μL) of volatile oil was diluted to 10 mL with n‐hexane for analysis.

### 4.3. Preparation of C_8_‐C_40_ n‐Alkane Mixed Reference Standard Solution

An accurately measured volume (1.0 mL) of the C_8_‐C_40_ n‐alkane mixed reference standard was transferred into separate 10 mL brown volumetric flasks and diluted to the calibration mark with n‐hexane and ethyl acetate, respectively. The resulting solutions were hermetically sealed with Parafilm and maintained at 4°C for subsequent analysis.

## 5. UPLC‐Q‐Exactive‐Plus‐Orbitrap‐MS Analysis Conditions

### 5.1. Instrumentation and Conditions

Chromatographic separation was performed on a Waters ACQUITY UPLC HSS T3 column (2.1 × 100 mm, 1.8 μm) at a flow rate of 0.2 mL/min. The injection volume was 10 μL. The mobile phase was composed of Solvent A (deionized water with 0.1% formic acid) and Solvent B (acetonitrile with 0.1% formic acid). The gradient program is shown in Table 1.

**TABLE 1:** Gradient program for qualitative analysis of fennel aqueous extract.

Time	Flow rate (mL/min)	A (%)	B (%)
0	0.2	100	0
10	0.2	70	30
25	0.2	60	40
30	0.2	50	50
40	0.2	30	70
45	0.2	0	100
60	0.2	0	100
70	0.2	100	0

Mass spectrometric data acquisition was performed using a Q Exactive Orbitrap high‐resolution mass spectrometer. The detection mode was Full MS‐ddMS^2^. Positive and negative ion modes were scanned simultaneously over an m/z range of 100–1500. The other parameters were set as follows: MS^1^ resolution, 70,000; MS^2^ resolution, 17,500; capillary voltage, 3.2 kV; capillary temperature, 320°C; auxiliary gas heater temperature, 350°C; sheath gas flow rate, 40 L/min; auxiliary gas flow rate, 15 L/min; AGC target, 1e6; Top N, 5; and stepped normalized collision energy (NCE), 30, 40, and 50.

### 5.2. Data Analysis and Identification of Compounds

Chromatographic peaks were identified by comparing retention times, precursor ions, and fragment ions with reference standards. Compounds with mzCloud (https://www.mz-cloud.com) and in‐house mzVault database scores greater than 80 were selected. Accurate molecular formulas were calculated based on exact mass (mass error < 5 ppm), with structures validated against predicted fragments. Final assignments were confirmed through literature review and cross‐referencing with PubChem, CNKI, and PubMed databases.

Furthermore, representative compounds from each chemical class were selected to elucidate their characteristic fragmentation pathways and diagnostic product ions based on high‐resolution MS/MS data and previously reported literature.

## 6. GC‐MS/MS Analysis Conditions

### 6.1. Instrumentation and Conditions

GC‐MS/MS analysis of the volatile oil was performed on an HP‐5MS capillary column (30 m × 0.25 mm × 0.25 μm). Helium was used as the carrier gas at a constant flow rate of 1.0 mL/min. The injection volume was 1 μL at an inlet temperature of 260°C with a split ratio of 10:1. Electron ionization (EI) was utilized at 70 eV. The ion source temperature was 250°C, and the solvent delay was 3 min. The scanning mass range was set from 40 to 650. The temperature program was as follows: starting at 50°C for 3 min, then ramping to 110°C at a rate of 3°C/min with a hold time of 2 min, followed by an increase to 240°C at a rate of 3°C/min.

### 6.2. Data Analysis and Identification of Compounds

The raw data files were imported into Qualitative Navigator B.08.00 software for further analysis. Peak integration and mass spectra extraction were performed using this software. The extracted mass spectra were compared to the National Institute of Standards and Technology (NIST) standard library for identification. In addition, the raw data were subjected to peak extraction and peak alignment. The compounds were identified by combining relevant literature and utilizing online databases. The Kovats retention index (KI) of the obtained volatile components was then determined from the NIST to further identify and verify these components.

The retention times of the C_8_‐C_40_ n‐alkane standard mixture were recorded under the aforementioned GC‐MS/MS conditions. The KI of the fennel volatile oil fraction was calculated according to the KI formula for linear temperature–programmed chromatography. Target compounds were identified by matching their experimental KI values against the NIST 20 mass spectral library and the NIST Chemistry WebBook database (https://webbook.nist.gov/chemistry/). Compounds with a deviation of less than 10 units between the experimental KI and the literature KI∗ values were considered for identification.

### 6.3. Statistical Analysis

All data were processed and summarized using WPS Office Excel 2025. The pie chart was generated using WPS Office Word 2025.

## 7. Results

### 7.1. UPLC‐Q‐Exactive‐Plus‐Orbitrap‐MS Analysis of Chemical Components From Fennel Aqueous Extraction

High‐resolution mass spectrometric data for the fennel aqueous extract were rapidly acquired using UPLC‐Q‐Exactive‐plus‐Orbitrap‐MS. A total of 87 compounds in the aqueous extract were systematically identified by UPLC‐Q‐Exactive‐plus‐Orbitrap‐MS in both positive and negative ion modes. The total ion chromatogram was analyzed by comparison with reference standards, interpretation of fragmentation patterns, and consultation of the mzCloud and mzVault databases (Figure 2 and Table 2). A total of 87 compounds were identified and classified from the aqueous extract of F. *vulgare* via UPLC‐MS analysis. These included 10 flavonoids, 11 phenolic acids, 3 coumarins, 1 stilbene, 14 alkaloids, 13 organic acids, 7 fatty acids, 10 amino acids, 6 nucleosides, 2 glycosides, 3 terpenoids, and 6 other components (containing 1 sugar alcohol). The relative proportions of the different compound classes are shown in Figure 3. Furthermore, representative compounds from each chemical class were selected as examples to analyze the possible fragmentation pathways and characteristic product ions, in conjunction with previously reported related literature and high‐resolution mass spectrometry fragment information.

**FIGURE 2:**
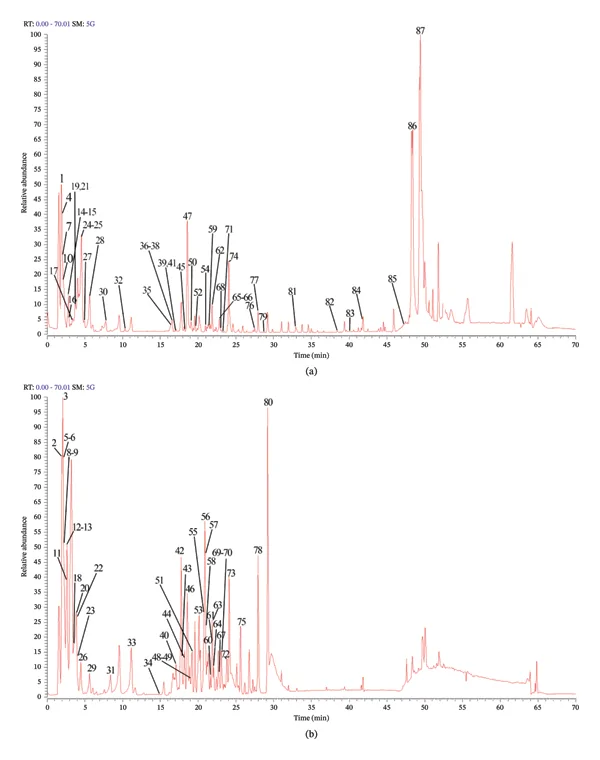
Total ion current chromatograms of fennel aqueous extract under positive (a) and negative (b) ion modes.

**TABLE 2:** Identification of chemical constituents of fennel aqueous extract by UPLC‐Q‐Exactive‐plus‐Orbitrap‐MS.

No.	RT (min)	Reference ion	m/z	Diff. (ppm)	Formula	Fragment ions (m/z)	Identification	Classification	References
1	1.826	[M + H]^+1^	104.107	0.08	C_5_H_13_NO	58.06549	Choline	Alkaloids	[18]
2	1.88	[M − H]^−1^	181.07186	0.55	C_6_H_14_O_6_	101.02432, 89.02431, 71.01373, 59.01378	D‐(−)‐Mannitol	Sugar alcohol	[19]
3	1.918	[M − H]^−1^	195.05093	−0.5	C_6_H_12_O_7_	195.05112	Gluconic acid	Organic acids	[20]
4	1.936	[M + H]^+1^	118.0862	−0.49	C_5_H_11_NO_2_	58.06549	Betaine	Alkaloids	[18]
5	1.963	[M − H]^−1^	191.05611	0.01	C_7_H_12_O_6_	191.05631, 87.00867	D‐(−)‐Quinic acid	Organic acids	[21]
6	1.988	[M − H]^−1^	341.10888	0.02	C_12_H_22_O_11_	341.11023, 179.05638. 161.04645, 119.03495	Sucrose	Glycosides	[22]
7	1.993	[M + H]^+1^	146.09238	−0.08	C_5_H_11_N_3_O_2_	128.08163, 104.07065, 87.04411, 86.06007	4‐Guanidinobutyric acid	Amino acids	[23]
8	2.009	[M − H]^−1^	173.009	−0.92	C_6_H_6_O_6_	85.02942, 129.01952	*trans-*Aconitic acid	Organic acids	[24]
9	2.041	[M − H]^−1^	111.00862	−1.36	C_5_H_4_O_3_	111.00871	2‐Furoic acid	Organic acids	[25]
10	2.128	[M + H]^+1^	130.04991	0.08	C_5_H_7_NO_3_	84.04449, 56.04990	L‐Pyroglutamic acid	Amino acids	[26]
11	2.486	[M − H]^−1^	341.10886	−0.04	C_12_H_22_O_11_	179.05644, 161.04524, 143.03503, 113.02438	α,α‐Trehalose	Glycosides	[27]
12	2.634	[M − H]^−1^	133.01416	−0.64	C_4_H_6_O_5_	115.00358, 72.99302, 71.01373	L‐(−)‐Malic acid	Organic acids	[28]
13	2.639	[M − H]^−1^	115.00352	−1.43	C_4_H_4_O_4_	71.01373	Fumaric acid	Organic acids	[24]
14	2.794	[M + H]^+1^	124.03935	0.39	C_6_H_5_NO_2_	78.03394, 80.04954, 106.02879, 124.03931	Nicotinic acid	Alkaloids	[29]
15	2.801	[M + H]^+1^	110.06007	0.19	C_6_H_7_NO	95.02390, 92.04947	3‐Hydroxy‐2‐methylpyridine	Alkaloids	[30]
16	3.015	[M + H]^+1^	137.04579	0.05	C_5_H_4_N_4_O	137.04575	Hypoxanthine	Alkaloids	[25]
17	3.296	[M + H]^+1^	130.04991	0.16	C_5_H_7_NO_3_	130.04987, 84.04449	D‐(+)‐Pyroglutamic acid	Amino acids	[31]
18	3.567	[M − H]^−1^	188.05653	0.32	C_7_H_11_NO_5_	144.06660, 170.04588, 102.05597	N‐Acetyl‐DL‐glutamic acid	Amino acids	[31]
19	3.68	[M + H]^+1^	182.08123	0.34	C_9_H_11_NO_3_	136.07567, 123.04405, 119.04914, 91.05428	L‐Tyrosine	Amino acids	[31]
20	3.694	[M − H]^−1^	243.06225	0.06	C_9_H_12_N_2_O_6_	200.05632, 152.03540, 111.02805, 110.02467, 66.03480	Uridine	Nucleosides	[19]
21	3.72	[M + H]^+1^	132.10194	0.25	C_6_H_13_NO_2_	86.09644, 69.07008	Isoleucine	Amino acids	[27]
22	3.847	[M − H]^−1^	117.01922	−0.91	C_4_H_6_O_4_	73.02940	Succinic acid	Organic acids	[28]
23	4.007	[M − H]^−1^	362.05098	0.44	C_10_H_14_N_5_O_8_P	133.01555	Guanosine monophosphate (GMP)	Nucleosides	[32]
24	4.479	[M + H]^+1^	136.06178	0.03	C_5_H_5_N_5_	136.06172, 119.03519, 92.02439	Adenine	Alkaloids	[20]
25	4.491	[M + H]^+1^	268.10388	−0.52	C_10_H_13_N_5_O_4_	136.06172, 119.03514	Adenosine	Nucleosides	[29]
26	4.505	[M − H]^−1^	161.04552	−0.15	C_6_H_10_O_5_	57.03447, 99.04508	3‐Hydroxy‐3‐methylglutaric acid	Organic acids	[24]
27	4.867	[M + H]^+1^	252.10876	−1.4	C_10_H_13_N_5_O_3_	252.10851, 99.04401	2′‐Deoxyadenosine	Nucleosides	[26]
28	5.576	[M + H]^+1^	152.05664	−0.3	C_5_H_5_N_5_O	135.03008, 110.03490	Guanine	Alkaloids	[33]
29	5.577	[M − H]^−1^	282.0844	−0.33	C_10_H_13_N_5_O_5_	107.03616	Guanosine	Nucleosides	[20]
30	7.735	[M + H]^+1^	166.08623	−0.17	C_9_H_11_NO_2_	149.05957, 120.08070, 79.05439, 93.06986, 103.05419	L‐Phenylalanine	Amino acids	[30]
31	8.36	[M − H]^−1^	137.02428	−0.94	C_7_H_6_O_3_	93.03444	Salicylic acid	Phenolic acids	[24]
32	10.32	[M + H]^+1^	127.05022	0.12	C_5_H_6_N_2_O_2_	110.02365	Thymine	Nucleosides	[34]
33	11.462	[M − H]^−1^	153.01928	−0.32	C_7_H_6_O_4_	109.02946	2,3‐Dihydroxybenzoic acid	Phenolic acids	[24]
34	14.919	[M − H]^−1^	353.08795	0.41	C_16_H_18_O_9_	—	1‐Caffeoylquinic acid	Phenolic acids	___
35	16.331	[M + H]^+1^	146.06006	0.13	C_9_H_7_NO	105.04512, 95.04964	4‐Hydroxyquinoline	Alkaloids	[27]
36	16.415	[M + H]^+1^	188.07055	−0.3	C_11_H_9_NO_2_	170.05994, 146.05992	Indole‐3‐acrylic acid	Alkaloids	[31]
37	16.424	[M + H]^+1^	205.09709	−0.18	C_11_H_12_N_2_O_2_	188.07050, 146.05995, 132.08081, 118.06505	L‐Tryptophan	Amino acids	[31]
38	16.558	[M + H]^+1^	146.06004	−0.06	C_9_H_7_NO	118.06509	8‐Hydroxyquinoline	Alkaloids	[35]
39	16.921	[M + H]^+1^	151.11176	0.09	C_10_H_14_O	123.11671	L‐(−)‐Carvone	Terpenoids	[31]
40	16.929	[M − H]^−1^	353.0878	−0.05	C_16_H_18_O_9_	353.08939, 191.05627, 179.03511	Chlorogenic acid	Phenolic acids	[25]
41	17.079	[M + H]^+1^	220.11787	−0.36	C_9_H_17_NO_5_	184.09674, 202.10716, 220.11743	Pantothenic acid	Organic acids	[36]
42	17.662	[M − H]^−1^	339.07217	−0.07	C_15_H_16_O_9_	339.07269	Esculin	Coumarins	[37]
43	17.885	[M − H]^−1^	175.06114	−0.33	C_7_H_12_O_5_	131.07143, 85.06582	2‐Isopropylmalic acid	Organic acids	[31]
44	18.083	[M − H]^−1^	193.05056	−0.21	C_10_H_10_O_4_	149.06078, 134.03735, 111.00879	Ferulic acid	Phenolic acids	[31]
45	18.337	[M + H]^+1^	190.04982	−2.76	C_10_H_7_NO_3_	116.04937, 144.04434	Kynurenic acid	Alkaloids	[38]
46	18.631	[M − H]^−1^	223.06115	−0.36	C_11_H_12_O_5_	179.07132, 223.06111	Sinapic acid	Phenolic acids	[20]
47	18.706	[M + H]^+1^	123.04398	−0.59	C_7_H_6_O_2_	105.04512, 95.04964	4‐Hydroxybenzaldehyde	Others	[27]
48	19.032	[M − H]^−1^	177.01929	−0.24	C_9_H_6_O_4_	89.03961, 105.03450, 133.02959	Esculetin	Coumarins	[39]
49	19.084	[M − H]^−1^	159.06616	−0.75	C_7_H_12_O_4_	115.07639, 97.06580	Pimelic acid	Organic acids	[31]
50	19.102	[M + H]^+1^	188.07054	−0.36	C_11_H_9_NO_2_	118.06509, 170.05998, 188.07054	*trans*‐3‐Indoleacrylic acid	Alkaloids	[33]
51	19.248	[M − H]^−1^	121.0294	−0.85	C_7_H_6_O_2_	121.02950, 93.03453	Benzoic acid	Phenolic acids	[40]
52	19.774	[M + H]^+1^	377.14544	−0.22	C_17_H_20_N_4_O_6_	243.08752, 200.08209, 172.08679, 99.04413	Riboflavin	Others	[31]
53	20.177	[M − H]^−1^	172.09788	−0.2	C_8_H_15_NO_3_	130.08728, 111.00878	N‐Acetyl‐L‐leucine	Amino acids	[31]
54	21.037	[M + H]^+1^	165.05462	−0.09	C_9_H_8_O_3_	119.05014	4‐Coumaric acid	Phenolic acids	[41]
55	21.238	[M − H]^−1^	577.15652	0.23	C_27_H_30_O_14_	433.11249, 415.10165, 397.09146, 367.08093, 337.07016, 313.07025, 283.05978	Vitexin rhamnoside	Flavonoids	[42]
56	21.284	[M − H]^−1^	206.08217	−0.45	C_11_H_13_NO_3_	164.07166, 147.04515	N‐Acetyl‐L‐phenylalanine	Amino acids	[30]
57	21.336	[M − H]^−1^	609.14624	0.07	C_27_H_30_O_16_	301.03430, 300.02765, 271.02490, 151.00362	Rutin	Flavonoids	[31]
58	21.384	[M − H]^−1^	165.0556	−0.65	C_9_H_10_O_3_	165.05559	3‐Phenyllactic acid	Phenolic acids	[43]
59	21.421	[M + H]^+1^	121.06476	−0.28	C_8_H_8_O	105.04476, 77.03866	Acetophenone	Others	[44]
60	21.433	[M − H]^−1^	173.0818	−0.79	C_8_H_14_O_4_	111.08149, 83.05016	Suberic acid	Organic acids	[31]
61	21.746	[M − H]^−1^	463.08858	0.56	C_21_H_20_O_12_	300.02765, 271.02490	Quercetin‐3β‐D‐glucoside	Flavonoids	[31]
62	21.843	[M + H]^+1^	303.0495	−1.53	C_15_H_10_O_7_	257.0441	Quercetin	Flavonoids	[45]
63	21.852	[M − H]^−1^	477.06753	−0.13	C_21_H_18_O_13_	151.00368, 178.99869	Quercetin 3‐O‐β‐D‐glucuronide	Flavonoids	[45]
64	22.162	[M − H]^−1^	593.15167	0.52	C_27_H_30_O_15_	285.04034, 284.03293, 255.03018	Kaempferol‐3‐O‐rutinoside	Flavonoids	[24]
65	22.709	[M + H]^+1^	336.12253	−1.51	C_20_H_17_NO_4_	320.09146, 292.09647	Epiberberine	Alkaloids	[46]
66	22.722	[M + H]^+1^	287.05471	−1.14	C_15_H_10_O_6_	153.01826, 121.02843	Kaempferol	Flavonoids	[47]
67	22.725	[M − H]^−1^	461.0728	0.09	C_21_H_18_O_12_	285.04068	Scutellarin	Flavonoids	[48]
68	22.948	[M + H]^+1^	317.06533	−0.79	C_16_H_12_O_7_	302.04187, 153.01817	Isorhamnetin	Flavonoids	[26]
69	23.04	[M − H]^−1^	609.183	0.72	C_28_H_34_O_15_	609.18311, 301.07202	Neohesperidin	Flavonoids	[49]
70	23.148	[M − H]^−1^	515.11978	0.54	C_25_H_24_O_12_	515.11957, 353.08798, 191.05617, 173.04550, 135.04517	4,5‐Dicaffeoylquinic acid	Phenolic acids	[37]
71	23.352	[M + H]^+1^	229.08579	−0.45	C_14_H_12_O_3_	211.07520, 193.06329	Resveratrol	Stilbenes	[30]
72	23.525	[M − H]^−1^	187.0976	0.08	C_9_H_16_O_4_	125.09710, 97.06579, 57.03443, 187.09766, 123.08149	Azelaic acid	Organic acids	[40]
73	23.981	[M − H]^−1^	137.0243	−0.81	C_7_H_6_O_3_	93.03445	3‐Hydroxybenzoic acid	Phenolic acids	[50]
74	24.058	[M + H]^+1^	336.1225	−1.59	C_20_H_17_NO_4_	292.09644	Berberine	Alkaloids	[51]
75	25.336	[M − H]^−1^	263.12892	0.2	C_15_H_20_O_4_	219.13918, 201.12866	(±)‐Abscisic acid	Terpenoids	[52]
76	27.59	[M + H]^+1^	187.03889	−0.41	C_11_H_6_O_3_	143.04915, 131.04913, 115.05423	Psoralen	Coumarins	[53]
77	27.914	[M + H]^+1^	293.21063	−1.67	C_18_H_28_O_3_	293.21063, 69.07014	12‐oxo Phytodienoic acid	Fatty acids	[26]
78	27.933	[M − H]^−1^	327.21764	−0.18	C_18_H_32_O_5_	327.21793, 229.14458, 97.06575	Corchorifatty acid F	Fatty acids	[54]
79	28.617	[M + H]^+1^	279.2317	−0.55	C_18_H_30_O_2_	173.13257, 243.21043	α‐Eleostearic acid	Fatty acids	[27]
80	29.192	[M − H]^−1^	329.2333	−0.15	C_18_H_34_O_5_	229.14456, 211.13403, 171.10269, 139.11278, 99.08144	(15Z)‐9,12,13‐Trihydroxy‐15‐octadecenoic acid	Fatty acids	[31]
81	33.098	[M + H]^+1^	233.15346	−0.64	C_15_H_20_O_2_	166.94531, 159.11671, 145.10114, 133.10110, 131.08554, 119.08552, 105.06989	Isoalantolactone	Terpenoids	[55]
82	38.453	[M + H]^+1^	193.08591	−0.03	C_11_H_12_O_3_	133.06477, 118.04127, 115.05424, 105.06992	Myristicin	Others	[56]
83	40.05	[M + H]^+1^	163.0753	−0.37	C_10_H_10_O_2_	91.0543	4‐Methoxycinnamaldehyde	Others	[57]
84	41.699	[M + H]^+1^	209.1171	−0.56	C_12_H_16_O_3_	181.08578	β‐Asarone	Others	[58]
85	47.1	[M + H]^+1^	279.23168	−0.64	C_18_H_30_O_2_	95.08558, 161.13239	α‐Linolenic acid	Fatty acids	[31]
86	48.269	[M + H]^+1^	282.27886	−0.99	C_18_H_35_NO	265.25226, 247.24208, 135.11687	Oleamide	Fatty acids	[33]
87	50.034	[M + H]^+1^	338.34119	−1.65	C_22_H_43_NO	69.07005, 97.10125	Erucamide	Fatty acids	[59]

**FIGURE 3:**
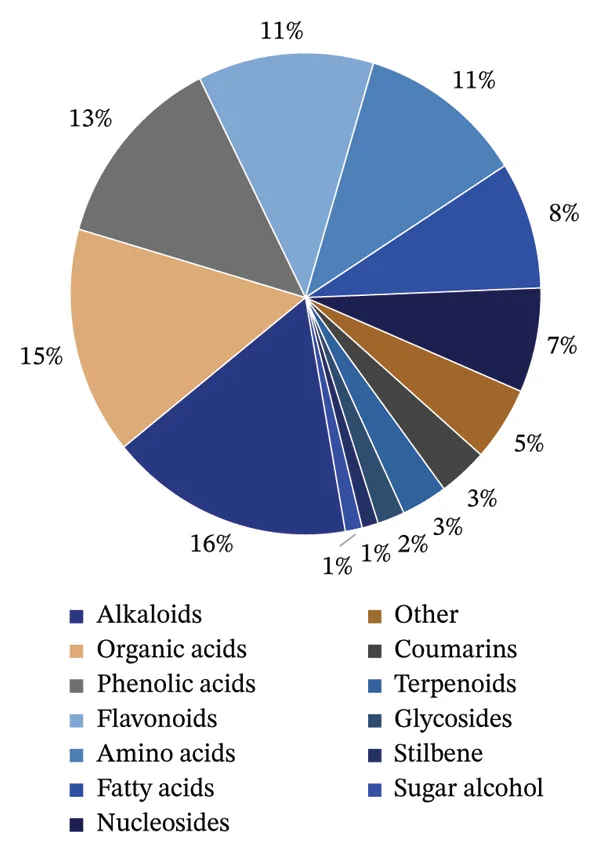
Types of compounds contained in fennel aqueous extract.

#### 7.1.1. Flavonoids

Flavonoids are plant polyphenols with a C_6_‐C_3_‐C_6_ skeleton (two benzene rings linked by a three‐carbon chain). In positive‐mode tandem MS, they form [M + H]^+^ ions and undergo characteristic CID fragmentations. The Retro–Diels–Alder (RDA) reaction is the hallmark cleavage of flavonoids, involving 1,3‐, 1,4‐, or 1,2‐ cleavage of the C‐ring to generate diagnostic A^+^ and B^+^ ions. Cross‐ring neutral losses include C_2_H_2_O (42 Da), C_4_H_4_O_2_ (84 Da), and C_3_O_2_ (68 Da), together with common losses of H_2_O (18 Da), CO (28 Da), and CO_2_ (44 Da). These distinct fragmentation fingerprints allow differentiation of flavonoid subclasses [60].

In positive ion mode, Compound **62** displayed a quasimolecular ion peak at m/z 303.04953 [M + H]^+^ (C_15_H_11_O_7_). Its MS/MS spectrum has two complementary fragmentation pathways: Pathway I involved sequential dehydration of the C‐ring, yielding fragment ions at m/z 285.03876 [M + H − H_2_O]^+^ and m/z 257.04410 [M + H − 2H_2_O]^+^, followed by the neutral loss of C_7_H_4_O (benzene ring with substituents) to produce m/z 153.01808 [M + H−2H_2_O − C_7_H_4_O]^+^, and subsequent O_2_ loss generating 121.02834 [M + H−2H_2_O−C_7_H_4_O−O_2_]^+^ [61]. Pathway II involved RDA cleavage of the C‐ring, producing the diagnostic fragment ion at m/z 153.01808 (B^+^ ion, C_7_H_5_O_4_^+^) along with its complementary A^+^ ion at m/z 165.01807 (C_8_H_5_O_4_^+^), consistent with the reported fragmentation behavior of quercetin [62]. In addition, an alternative cleavage involving the neutral loss of C_7_H_6_O_3_ (122 Da) was observed, generating the fragment ion at m/z 165.01807. The detailed MS/MS spectrum and proposed fragmentation pathways of Compound **62** are illustrated in Figure 4.

**FIGURE 4:**
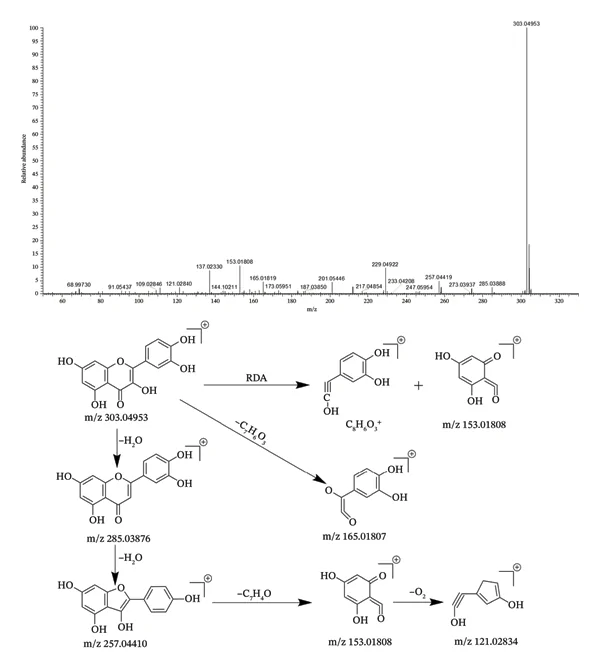
Secondary mass spectra and possible fragmentation pattern of quercetin.

In negative ion mode, Compound **57** displayed a quasimolecular ion peak at m/z 609.14661 [M − H]^−^ (C_27_H_29_O_16_). Its MS/MS spectrum revealed a dominant glycosidic bond cleavage pathway involving the neutral loss of the rutinose moiety (C_12_H_20_O_10_, 308 Da) to generate the quercetin aglycone ion at m/z 301.03430 [M − H − C_12_H_20_O_10_]^-^ [63]. The aglycone subsequently underwent three distinct fragmentation routes: decarboxylation with simultaneous hydrogen rearrangement, producing m/z 255.03006 [M − H − C_12_H_20_O_10_‐HCOOH]^-^; RDA cleavage of the C‐ring with the neutral loss of C_7_H_6_O_2_ (122 Da), yielding m/z 178.99854 [M − H − C_12_H_20_O_10_ − C_7_H_6_O_2_]^−^; and cross‐ring cleavage involving the loss of C_8_H_6_O_3_ (150 Da) to form m/z 151.00362 [M − H − C_12_H_20_O_10_ − C_8_H_6_O_3_]^−^. The latter two fragments (m/z 178 and m/z 151) represent characteristic A‐ring– and B‐ring–derived ions, consistent with the reported fragmentation behavior of quercetin aglycone [64]. The detailed MS/MS spectrum and proposed fragmentation pathways of Compound **57** are illustrated in Figure 5.

**FIGURE 5:**
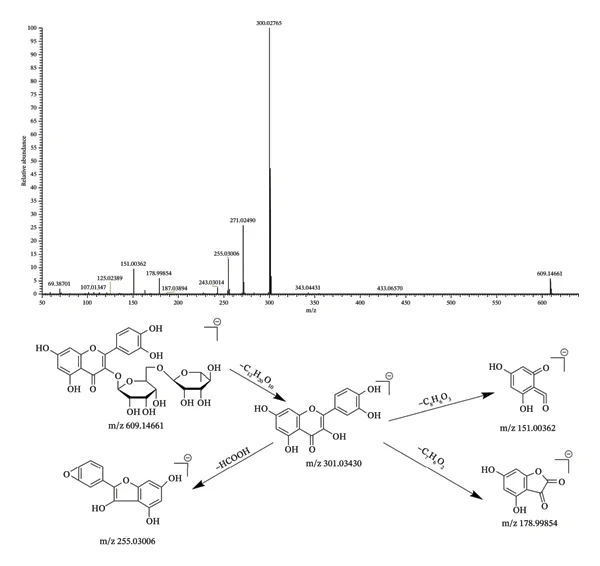
Secondary mass spectra and possible fragmentation pattern of rutin.

In positive ion mode, Compound **66** displayed a quasimolecular ion peak at m/z 287.05484 [M + H]^+^ (C_15_H_11_O_6_). Its MS/MS spectrum revealed two major fragmentation pathways: Pathway I involved RDA cleavage of the C‐ring with the neutral loss of C_4_H_4_O_4_ (116 Da), generating the diagnostic A^+^ fragment ion at m/z 171.04381, which subsequently underwent dehydration to produce m/z 153.01826 [M + H − C_4_H_4_O_4_ − H_2_O]^+^. Pathway II comprised consecutive small‐molecule losses from the [M + H]^+^ precursor, including CO elimination (28 Da) to yield m/z 259.05841 [M + H − CO]^+^, followed by H_2_O loss (18 Da) to form m/z 241.04942 [M + H − CO − H_2_O]^+^. Further fragmentation of the m/z 259 ion via phenolic neutral loss of C_6_H_6_O (94 Da) produced m/z 165.01830 [M + H − CO − C_6_H_6_O]^+^ [65]. The detailed MS/MS spectrum and proposed fragmentation pathways of Compound **66** are illustrated in Figure 6.

**FIGURE 6:**
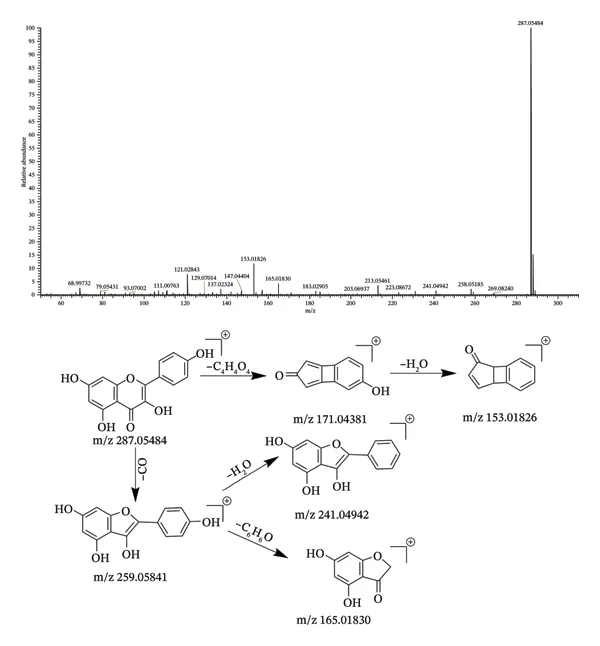
Secondary mass spectra and possible fragmentation pattern of kaempferol.

#### 7.1.2. Alkaloids

Alkaloids are nitrogen‐containing basic organic compounds with diverse structures (indole, quinoline, isoquinoline, tropane, purine, etc.). In positive‐ion mode, they readily form [M + H]^+^ quasimolecular ions. Under high‐energy collision, characteristic fragmentations include nitrogen heterocycle ring‐opening (with losses of NH_3_, CH_3_NH_2_, or C_2_H_5_NH_2_); C‐N bond cleavage; and neutral losses of H_2_O, CO, and CO_2_. N‐methyl–substituted alkaloids tend to lose CH_3_ radicals, while methoxy‐substituted ones lose CH_3_O or CH_3_OH. For glycosylated alkaloids, glycosidic bond cleavage occurs (glucose 162 Da, rhamnose 146 Da, etc.), yielding aglycone fragments [66]. In positive ion mode, Compound **1** displayed a quasimolecular ion peak at m/z 104.10694 [M]^+^ (C_5_H_14_N^+^). Its MS/MS spectrum yielded a characteristic fragment ion at m/z 60.08112 [M − C_2_H_4_O]^+^. The fragmentation pathway involves a characteristic neutral loss of acetaldehyde (C_2_H_4_O, 44 Da) from the quaternary ammonium precursor, generating the trimethylammonium ion (C_3_H_10_N^+^, m/z 60) [67]. The detailed MS/MS spectrum and proposed fragmentation pathway of Compound **1** are illustrated in Figure 7.

**FIGURE 7:**
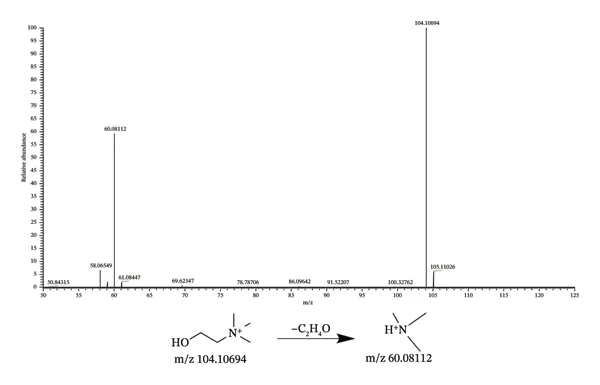
Secondary mass spectra and possible fragmentation pattern of choline.

#### 7.1.3. Amino Acids

Amino acids are organic compounds containing both amino and carboxyl functional groups. In tandem mass spectrometry analysis, these compounds typically undergo characteristic neutral losses from their protonated molecular ions, including H_2_O (18 Da), CO_2_ (44 Da), and NH_3_ (17 Da) [68]. In positive ion mode, Compound **30** displayed a quasimolecular ion peak at m/z 166.08614 [M + H]^+^ (C_9_H_12_NO_2_). Its MS/MS spectrum revealed two primary competitive fragmentation pathways: Pathway I involved the characteristic loss of the carboxyl group as formic acid (HCOOH, 46 Da) from the protonated amino acid, generating the benzylammonium‐type fragment ion at m/z 120.08070 [M + H − COOH]^+^ (C_8_H_10_N^+^), which subsequently underwent ammonia elimination (NH_3_, 17 Da) to produce the styryl cation at m/z 103.05419 [M + H − COOH − NH_3_]^+^ (C_8_H_7_^+^) [69, 70]. Pathway II proceeded via direct ammonia loss from the protonated molecular ion, yielding m/z 149.05957 [M + H − NH_3_]^+^ (C_9_H_9_O_2_^+^), corresponding to the deamination product retaining the carboxyl functionality. The formation of m/z 103 through sequential decarboxylation and deamination represents a diagnostic fragmentation pattern for aromatic amino acids, reflecting the stability of the resulting styryl cation stabilized by phenyl conjugation [70]. The detailed MS/MS spectrum and proposed fragmentation pathways of Compound **30** are illustrated in Figure 8.

**FIGURE 8:**
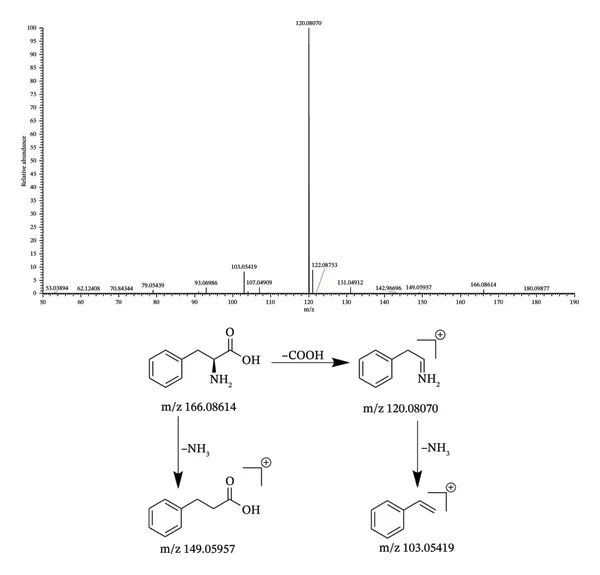
Secondary mass spectra and possible fragmentation pattern of L‐phenylalanine.

#### 7.1.4. Coumarin

Coumarins are natural benzopyrone‐fused phenylpropanoids. In negative‐ion tandem mass spectrometry, they typically form deprotonated quasimolecular ions [M − H]^−^ and undergo characteristic fragmentations: lactone ring opening with neutral losses of CO (28 Da) and CO_2_ (44 Da) and C‐O bond cleavage in the pyrone ring yielding phenolic fragments. Additional common losses include H_2_O (18 Da), C_2_H_2_O (42 Da), and the isoprenyl group (56 Da) when present [71]. In negative ion mode, Compound **48** displayed a quasimolecular ion peak at m/z 177.01942 [M − H]^−^ (C_9_H_5_O_4_). Its MS/MS spectrum revealed two competitive primary fragmentation pathways originating from the deprotonated coumarin nucleus: Pathway I involved initial CO elimination (28 Da) from the lactone carbonyl, generating m/z 149.02460 [M − H − CO]^−^ (C_8_H_5_O_3_^−^), which subsequently underwent decarboxylation (CO_2_ loss, 44 Da) to produce m/z 105.03450 [M − H − CO − CO_2_]^−^ (C_7_H_5_O^−^) [72, 73]. Pathway II proceeded via direct decarboxylation of the precursor ion, yielding m/z 133.02959 [M − H − CO_2_]^−^ (C_8_H_5_O_2_^−^), which further eliminated a second CO_2_ molecule to form m/z 89.03961 [M − H − 2CO_2_]^−^ (C_6_H_5_O^−^) [73]. These consecutive neutral losses of CO and CO_2_ from the pyrone ring are diagnostic fragmentation patterns for hydroxycoumarins, reflecting the sequential breakdown of the α‐pyrone lactone structure under CID conditions. The coexistence of both pathways indicates competitive fragmentation at the lactone carbonyl versus the ring carboxylate positions. The detailed MS/MS spectrum and proposed fragmentation pathways of Compound **48** are illustrated in Figure 9.

**FIGURE 9:**
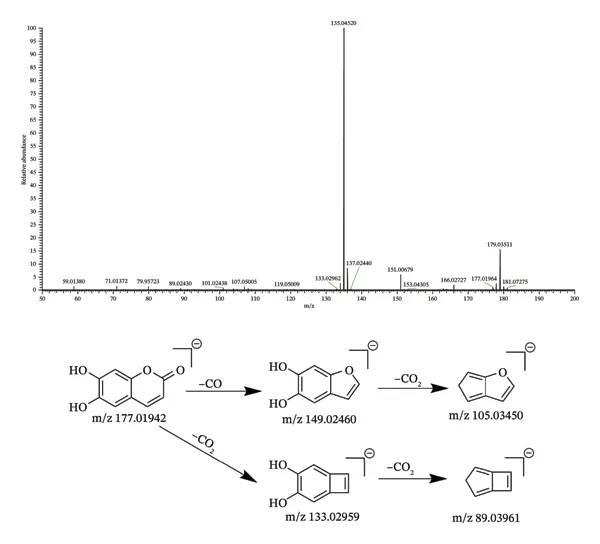
Secondary mass spectra and possible fragmentation pattern of esculetin.

#### 7.1.5. Organic Acids

Organic acids, containing acidic functional groups (e.g., carboxyl and phenolic hydroxyl), predominantly form deprotonated quasimolecular ions [M − H]^−^ in negative ion mode. Under high‐energy collision, they typically undergo characteristic neutral losses of H_2_O (18 Da), CO_2_ (44 Da), and COOH (45 Da), yielding diagnostic fragment ions [74]. Its MS/MS spectrum revealed two competitive primary fragmentation pathways: Pathway I involved intramolecular dehydration between the α‐hydroxyl group and adjacent hydrogen, generating the fumaric acid (or maleic acid) anion at m/z 115.00358 [M − H − H_2_O]^−^ (C_4_H_3_O_4_^−^), which subsequently underwent decarboxylation (CO_2_ loss, 44 Da) to produce the vinyl carboxylate anion at m/z 71.01373 [M − H − H_2_O − CO_2_]^−^ (C_3_H_3_O^−)^. Pathway II proceeded via direct decarboxylation of the precursor ion, yielding m/z 89.02435 [M − H − CO_2_]^−^ (C_3_H_5_O_3_^−^), corresponding to the glycolic acid anion or its enolate equivalent. The sequential neutral losses of H_2_O and CO_2_ from hydroxycarboxylic acids are diagnostic fragmentation patterns for aliphatic organic acids, reflecting the facile dehydration of β‐hydroxy acids followed by decarboxylation of the resulting unsaturated carboxylate under CID conditions. Based on comparison with the reference standard [42], Compound **12** was identified as L‐(−)‐malic acid. The detailed MS/MS spectrum and proposed fragmentation pathways are illustrated in Figure 10.

**FIGURE 10:**
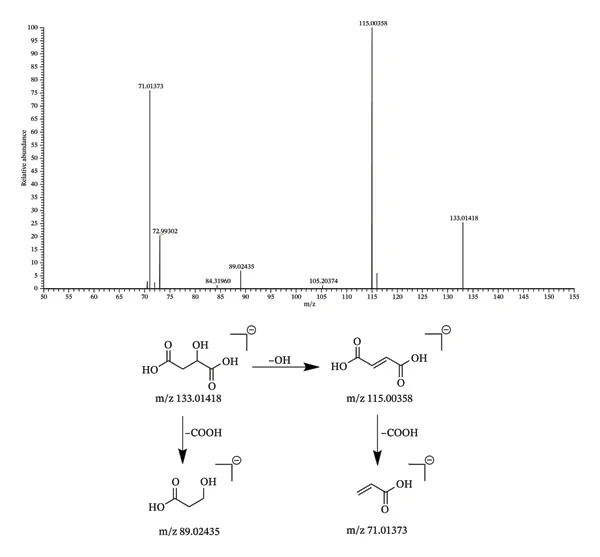
Secondary mass spectra and possible fragmentation pattern of L‐(−)‐malic acid.

#### 7.1.6. Phenolic Acid

Phenolic acids are aromatic organic acids containing phenolic hydroxyl and carboxyl groups, including hydroxybenzoic (C_6_‐C_1_) and hydroxycinnamic (C_6_‐C_3_) subclasses. In negative ion mode tandem mass spectrometry, they readily form [M − H]‐ quasimolecular ions and undergo characteristic fragmentations: neutral loss of CO_2_ (44 Da) generating phenolic fragments, C‐C bond cleavage with COOH loss (45 Da), and H_2_O elimination (18 Da) favored by ortho‐positioned groups through intramolecular hydrogen bonding. Hydroxycinnamic acids additionally produce benzoyl (m/z 105) or cinnamoyl (m/z 131) fragments via side chain cleavage. Methoxy‐substituted compounds lose CH_3_ (15 Da) or CH_3_O (31 Da), while feruloyl‐containing compounds show characteristic feruloyl moiety loss (176 Da) [75]. In negative ion mode, Compound **40** displayed a quasimolecular ion peak at m/z 353.08939 [M − H]^−^ (C_16_H_17_O_9_) [76]. Its MS/MS spectrum revealed two primary fragmentation pathways characteristic of caffeoylquinic acid esters: Pathway I involved ester bond cleavage with charge retention on the caffeoyl moiety, generating the deprotonated caffeic acid anion at m/z 179.03511 [M − H − C_7_H_10_O_5_]^−^ (C9H7O4^−^), which subsequently underwent decarboxylation (CO_2_ loss, 44 Da) to produce the dihydroxystyryl anion at m/z 135.04520 [M − H − C_7_H_10_O_5_ − CO_2_]^−^ (C_8_H_7_O_2_^−^) [77]. Pathway II proceeded via ester bond cleavage with charge retention on the quinic acid moiety, yielding the deprotonated quinic acid anion at m/z 191.05627 [M − H − C_9_H_6_O_3_]^−^ (C_7_H_11_O_6_^−^), which subsequently underwent two distinct secondary fragmentation routes: sequential dehydration and decarboxylation to form m/z 111.04500 [M − H − C_9_H_6_O_3_ − H_2_O − CO_2_]^−^ (C_6_H_7_O_2_^−^), and dehydration followed by C‐C bond cleavage with ethylene (C2H4) elimination to produce the dihydroxycyclopentadienyl anion at m/z 85.02943 [M − H − C_9_H_6_O_3_ − C_2_H_4_O_2_]^−^ (C_4_H_5_O_2_^−^). The competitive formation of m/z 179 (caffeoyl pathway) and m/z 191 (quinic acid pathway) represents a diagnostic fragmentation pattern for chlorogenic acid isomers, allowing differentiation based on the relative abundance ratios of these two complementary ions [77, 78]. The detailed MS/MS spectrum and proposed fragmentation pathways of Compound **40** are illustrated in Figure 11.

**FIGURE 11:**
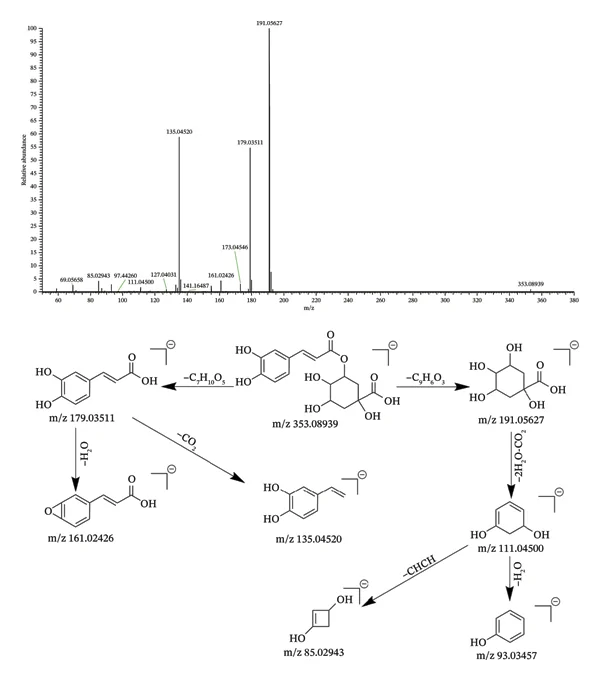
Secondary mass spectra and possible fragmentation pattern of chlorogenic acid.

In negative ion mode, Compound **44** displayed a quasimolecular ion peak at m/z 193.05055 [M − H]^-^ (C_10_H_9_O_4_). Its MS/MS spectrum revealed two competitive primary fragmentation pathways characteristic of methoxycinnamic acids: Pathway I involved homolytic cleavage of the O‐CH_3_ bond with loss of a methyl radical (CH_3_, 15 Da), generating the demethylated phenoxide anion at m/z 178.02721 [M − H − CH3]^−^ (C_9_H_6_O_4_^−^) [78], which subsequently underwent decarboxylation (CO_2_ loss, 44 Da) to produce the dihydroxystyryl anion at m/z 134.03735 [M − H − CO_2_ − CH_3_]^−^ (C_8_H_6_O_2_^−^) [79]. Pathway II proceeded via direct decarboxylation of the precursor ion, yielding the methoxystyryl phenoxide anion at m/z 149.06078 [M − H − CO_2_]^−^ (C_9_H_9_O_2_^−^) [78]. The coexistence of methyl radical loss and CO_2_ elimination reflects the competing fragmentation tendencies of the methoxy‐substituted aromatic ring versus the α, β‐unsaturated carboxylic acid functionality. The sequential neutral losses of CH_3_ and CO_2_ from m/z 178 to form m/z 134 represent a diagnostic fragmentation pattern for ferulic acid and related methoxycinnamic acids, allowing differentiation from caffeic acid [79]. The detailed MS/MS spectrum and proposed fragmentation pathways of Compound **44** are illustrated in Figure 12.

**FIGURE 12:**
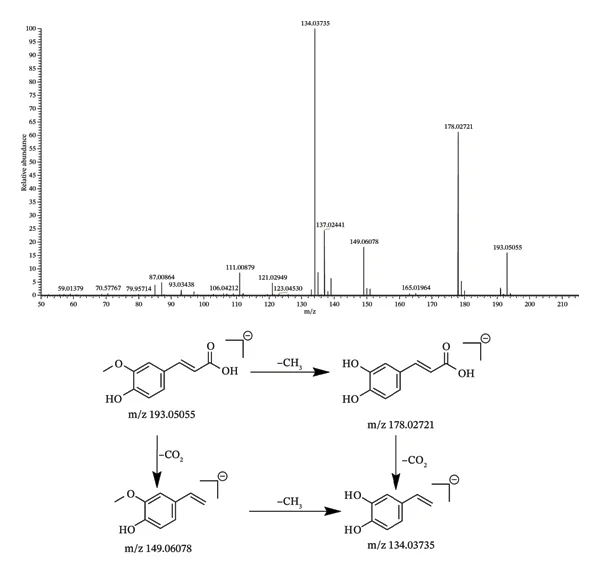
Secondary mass spectra and possible fragmentation pattern of ferulic acid.

### 7.2. GC‐MS/MS Analysis of Volatile Oil From Fennel

Under the aforementioned GC‐MS/MS conditions, the retention times of a C_8_‐C_40_ n‐alkane mixture were recorded. Samples were injected under GC‐MS/MS conditions, with n‐hexane and ethyl acetate used successively as solvents for the detection of volatile constituents in fennel. Representative total‐ion‐current (TIC) chromatograms of the fennel volatile oils obtained with n‐hexane and ethyl acetate as respective solvents are shown in Figure 13. The relative contents of individual components were calculated by peak‐area normalization. Components with relative abundances exceeding 0.01% were subjected to qualitative analysis. For each well‐resolved chromatographic peak, the KI of the volatile oil was calculated according to the linear temperature–programmed KI equation. Target compounds were identified by matching their experimental KI values against the NIST 20 spectral library and the NIST Chemistry WebBook database; only hits with reference KI∗ values differing by ≤ 10 units from the measured value were accepted. Detailed results are summarized in Tables 3 and 4.

**FIGURE 13:**
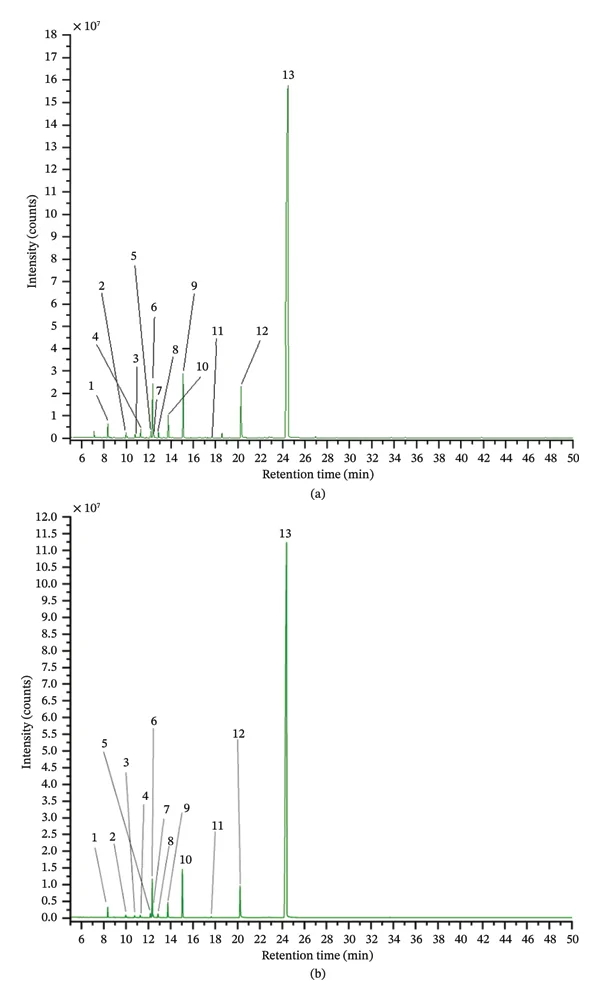
Total ion chromatogram obtained from the GC‐MS/MS analysis of the volatile oil components of fennel: using n‐hexane as the solvent (a) and using ethyl acetate as the solvent (b). X‐axis: retention time (min); Y‐axis: intensity (counts × 10^7^).

**TABLE 3:** Identification of chemical constituents of volatile oil components in fennel by GC‐MS/MS (n‐hexane).

No.	*t*_*R*_/min	Compound	CAS no.	Molecular formula	Molecular weight	Relative amount (%)	Classification	KI	KI^∗^
1	8.364	3‐Thujene	2867‐05‐2	C_10_H_16_	136.23	0.84	Monoterpene	938	933
2	9.984	Sabinene	3387‐41‐5	C_10_H_16_	136.23	0.34	Monoterpene	974	973
3	10.789	β‐Pinene	127‐91‐3	C_10_H_16_	136.23	0.25	Monoterpene	992	981
4	11.295	α‐Phellandrene	99‐83‐2	C_10_H_16_	136.23	0.57	Monoterpene	1003	1002
5	12.199	p‐Cymene	99‐87‐6	C_10_H_14_	136.23	0.45	Monoterpene	1022	1023
6	12.371	Limonene	138‐86‐3	C_10_H_16_	136.23	3.68	Monoterpene	1026	1027
7	12.464	1,8‐Cineole	470‐82‐6	C_10_H_18_O	154.25	0.21	Monoterpene	1028	1028
8	12.886	*trans*‐β‐Ocimene	3779‐61‐1	C_10_H_16_	136.23	0.39	Monoterpene	1037	1047
9	13.761	γ‐Terpinene	99‐85‐4	C_10_H_16_	136.23	1.62	Monoterpene	1057	1057
10	15.086	Fenchone	126‐21‐6	C_10_H_16_O	152.23	4.8	Monoterpene	1085	1085
11	17.665	Camphor	76‐22‐2	C_10_H_16_O	152.23	0.11	Monoterpene	1142	1143
12	20.262	Estragole	140‐67‐0	C_10_H_12_O	148.20	4.4	Phenylpropanoid	1199	1197
13	24.495	Anethole	104‐46‐1	C_10_H_12_O	148.20	80.62	Phenylpropanoid	1291	1290

*Note:* KI denotes the experimentally determined Kovats index of the target compound, whereas KI^∗^ represents its literature‐reported value.

**TABLE 4:** Identification of chemical constituents of volatile oil components in fennel by GC‐MS/MS (ethyl acetate).

No.	*t*_*R*_/min	Compound	CAS no.	Molecular formula	Molecular weight	Relative amount (%)	Classification	KI	KI^∗^
1	8.359	3‐Thujene	2867‐05‐2	C_10_H_16_	136.23	0.8	Monoterpene	938	933
2	9.975	Sabinene	3387‐41‐5	C_10_H_16_	136.23	0.21	Monoterpene	974	973
3	10.775	β‐Pinene	127‐91‐3	C_10_H_16_	136.23	0.22	Monoterpene	991	981
4	11.276	α‐Phellandrene	99‐83‐2	C_10_H_16_	136.23	0.21	Monoterpene	1002	1002
5	12.183	p‐Cymene	99‐87‐6	C_10_H_14_	136.23	0.38	Monoterpene	1022	1023
6	12.352	Limonene	138‐86‐3	C_10_H_16_	136.23	3.43	Monoterpene	1026	1027
7	12.45	1,8‐Cineole	470‐82‐6	C_10_H_18_O	154.25	0.22	Monoterpene	1028	1028
8	12.862	*trans*‐β‐Ocimene	3779‐61‐1	C_10_H_16_	136.23	0.34	Monoterpene	1037	1047
9	13.741	γ‐Terpinene	99‐85‐4	C_10_H_16_	136.23	1.42	Monoterpene	1056	1057
10	15.058	Fenchone	126‐21‐6	C_10_H_16_O	152.23	4.67	Monoterpene	1085	1085
11	17.636	Camphor	76‐22‐2	C_10_H_16_O	152.23	0.11	Monoterpene	1141	1143
12	20.234	Estragole	140‐67‐0	C_10_H_12_O	148.20	3.64	Phenylpropanoid	1198	1197
13	24.403	Anethole	104‐46‐1	C_10_H_12_O	148.20	83.45	Phenylpropanoid	1289	1290

*Note:* KI denotes the experimentally determined Kovats index of the target compound, whereas KI^∗^ represents its literature‐reported value.

A total of 13 constituents were recognized in the n‐hexane extract, representing 99.10% of the TIC. These comprised 11 monoterpenes (3‐thujene, sabinene, β‐pinene, α‐phellandrene, p‐cymene, limonene, *trans*‐β‐ocimene, γ‐terpinene, 1,8‐cineole, fenchone, and camphor) and 2 phenylpropanoids (estragole and anethole).

The ethyl acetate extract furnished 13 identified peaks, accounting for 99.10% of the volatiles. The chemical profile was similar. Overall, the pooled data demonstrate that fennel volatile oil is dominated by monoterpenes, followed by monoterpenoids and phenylpropanoids, as shown in Figures 13 and 14 and Tables 3 and 4.

**FIGURE 14:**
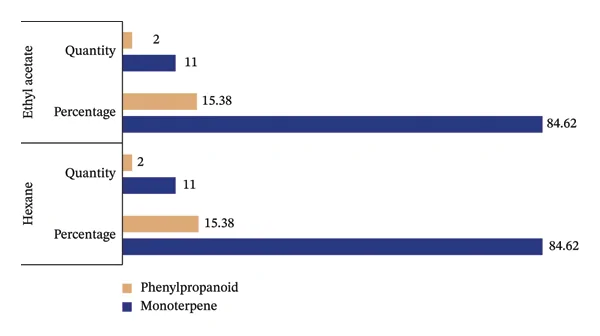
Bar chart showing the quantity and percentage of components in fennel volatile oil (n‐hexane and ethyl acetate).

## 8. Discussion

In recent years, UPLC‐Q‐Exactive‐plus‐Orbitrap‐MS and GC‐MS/MS have been increasingly employed for the comprehensive characterization of herbal medicines, bridging traditional knowledge and modern phytochemistry [80–83]. In this study, these complementary techniques were integrated for the first time to systematically profile the components of both the aqueous extract and volatile oil of fennel (*Foeniculum vulgare* Mill.).

### 8.1. Volatile Oil Analysis

GC‐MS/MS identified 13 volatile components accounting for 99.10% of the total content, dominated by phenylpropanoids (*trans*‐anethole and estragole) and oxygenated monoterpenes (fenchone, 1,8‐cineole, and camphor), alongside monoterpenes (limonene, sabinene, β‐pinene, α‐phellandrene, γ‐terpinene, 3‐thujene, p‐cymene, and *trans*‐β‐ocimene). Compared to literature reports [14, 84, 85], α‐pinene, myrcene, 4‐terpineol, α‐terpineol, terpinyl acetate, eugenol, and methyl chavicol were absent from the present sample, likely due to variations in geographical origin, harvest season, extraction method, or chromatographic conditions.

The results revealed that anethole was the most abundant constituent in the fennel volatile oil analyzed by GC‐MS/MS. However, this method alone could not determine the specific stereochemical configuration of anethole. *Trans*‐anethole was confirmed as the predominant constituent by matching its retention time with that of an authenticated reference standard under identical GC conditions, consistent with its designation as a quality control marker in the Chinese Pharmacopoeia [7, 86, 87]. As the signature aroma‐active component of fennel volatile oil, *trans*‐anethole has demonstrated cardioprotective, neuroprotective, and anti‐inflammatory activities [88–90], supporting its role as a lead compound for pharmacological investigation.

### 8.2. Aqueous Extract Analysis

UPLC‐Q‐Exactive‐plus‐Orbitrap‐MS identified 87 compounds, among which phenolic acids and flavonoids were the predominant classes [4, 15]. These included 10 flavonoids, 11 phenolic acids, 3 coumarins, 1 stilbene, 14 alkaloids, 13 organic acids, 7 fatty acids, 10 amino acids, 6 nucleosides, 2 glycosides, 3 terpenoids, and 6 other components (including 1 sugar alcohol).

Representative phenolic acids included ferulic acid, chlorogenic acid, salicylic acid, and 4,5‐dicaffeoylquinic acid. Chlorogenic acid, a caffeoylquinic acid derivative, is recognized as a bioactive marker in herbal medicines with established anti‐inflammatory, antimicrobial, and hypoglycemic properties [91, 92]. Furthermore, these phenolic compounds, particularly chlorogenic acid, have been demonstrated to possess significant antioxidant capacity through multiple in vitro assays [14]. Flavonoids existed as both aglycones (quercetin, kaempferol, and isorhamnetin) and glycosides (quercetin‐3‐O‐β‐D‐glucuronide, rutin, scutellarin, neohesperidin, and kaempferol‐3‐O‐rutinoside), the latter showing significant anti‐inflammatory potential [93].

However, several limitations should be acknowledged. First, the absence of certain volatile components relative to literature reports warrants further optimization of extraction and detection parameters to improve coverage. Second, UPLC‐Q‐Exactive‐plus‐Orbitrap‐MS alone could not determine absolute configurations or distinguish positional isomers (e.g., chlorogenic acid vs. cryptochlorogenic acid) due to the lack of chiral separation capability and authenticated reference standards [77]. Future studies employing chiral chromatography or co‐injection with reference standards are warranted to confirm stereochemical identities and elucidate structure–activity relationships. Third, although phenolic acids and flavonoids are not exclusive to fennel, their comprehensive profiling in this study provides reliable reference data for quality control and holistic evaluation.

This study represents the first simultaneous application of UPLC‐Q‐Exactive‐plus‐Orbitrap‐MS and GC‐MS/MS for integrated chemical profiling of both polar and nonpolar fractions of fennel. By establishing this dual‐platform methodology, we achieved complete chemical characterization across polarity gradients, transitioning from single‐marker analysis toward holistic phytochemical profiling. These findings provide a robust foundation for elucidating the pharmacodynamic material basis of fennel and support its continued development as both a pharmaceutical resource and functional food ingredient.

## 9. Conclusion

This study established an integrated analytical platform combining UPLC‐Q‐Exactive‐plus‐Orbitrap‐MS and GC‐MS/MS for the comprehensive chemical characterization of both aqueous extract and volatile oil of *Foeniculum vulgare*. A total of 87 polar metabolites and 13 volatile components were identified, revealing phenolic acids and flavonoids as the predominant constituents in the aqueous extract, and *trans-*anethole as the major volatile marker. These findings advance the quality control paradigm of fennel from single‐marker analysis toward holistic phytochemical profiling and provide a robust chemical basis for elucidating its pharmacodynamic properties. Future studies incorporating authenticated reference standards are warranted to confirm tentative identifications and further investigate structure–activity relationships of the identified constituents.

## Author Contributions

Nier Wu, Junni Qi, Xinxin Jin, Chula Sa, and Yan Zhang contributed significantly to query papers and paper analysis. Nier Wu organized the data and wrote the manuscript. Chula Sa and Yan Zhang helped perform the analysis with constructive discussions. All authors contributed to the study’s conception and design.

## Funding

The authors disclosed receipt of the following financial support for the research, authorship, and/or publication of this article: The Traditional Chinese Medicine “First‐Class Discipline Construction” Special Research Project (Grant no. YLXKZX‐NYD‐001) and the Inner Mongolia Medical University “First‐Class Discipline” Construction Innovation Team Project (Grant no. 2024MYYLXK006).

## Conflicts of Interest

The authors declare no conflicts of interest.

## Data Availability

The data used to support the findings of this study are included within the article.
